# Treatment of gorham-stout disease with bisphosphonates and total hip arthroplasty: A case report

**DOI:** 10.3389/fsurg.2023.1078869

**Published:** 2023-01-30

**Authors:** LiQian Zhang, JianJian Li, Fei Yao, YiHao Chen, Shuo Zhang, Hao Lv, JueHua Jing

**Affiliations:** ^1^ Department of Orthopaedics & Joint Reconstruction Surgery, The Second Hospital of Anhui Medical University, Anhui Medical University, Hefei, China; ^2^ Institute of Orthopaedics, Research Center for Translational Medicine, The Second Hospital of Anhui Medical University, Anhui Medical University, Hefei, China

**Keywords:** gorham-Stout disease, bisphosphonates, arthroplasty, hip joint, histopathological, radiological features

## Abstract

**Background:**

Gorham-Stout disease (GSD) is a rare osteolytic disease with unknown etiology, varied clinical manifestations and unpredictable prognosis. This disease is characterized by progressive massive local osteolysis and resorption caused by intraosseous lymphatic vessel structure and thin-walled vascular proliferation. The diagnosis of GSD has not yet formed a uniform standard, but the combination of clinical manifestations, radiological features and unique histopathological examinations and excluding other diseases contribute to early diagnosis. Although medical therapy, radiotherapy and surgical interventions or combinations have been used for the treatment of GSD, there is currently still no recommended standardized treatment regimen.

**Case report:**

This paper presents a case of a previously healthy 70-year-old man presented with a 10-year history of severe right hip pain and progressive walking disorder of the lower limbs. Based on the patient's clear clinical presentation, unique radiological features, and histological findings, a diagnosis of GSD was made with the exclusion of other potential diseases. The patient was treated with bisphosphonates to slow the progression of the disease followed by total hip arthroplasty to help restore walking function. At the 3-year follow-up, the patient returned to normal walking and no recurrence was observed.

**Conclusion:**

Bisphosphonates combined with total hip arthroplasty may be an effective method for the treatment of severe GSD in the hip joint.

## Introduction

Gorham-Stout disease (GSD), also termed idiopathic massive osteolysis, vanishing bone disease, phantom bone disease, acute spontaneous absorption of bone, and Gorham disease, is an exceedingly rare osteolytic disease characterized by progressive massive local osteolysis and resorption caused by spontaneous intraosseous lymphatic vessel structure and thin-walled vascular proliferation (1–3). At present, approximately 400 cases have been reported in the literature. Due to the low incidence and high misdiagnosis rate of GSD, the cases reported in the literature are case reports and small sample series cases (4). Although some researchers have conducted the cytology and molecular studies, the etiology and pathogenesis of GSD are still unclear (4, 5). The incidence of GSD does not differ significantly among genders, ages, regions, or ethnicities, although most cases occur in children as young as 1 month old and young adults under 40 years old (4, 6). In elderly patients, it often predicts a poor prognosis.

In most cases, GSD usually occurs in a single bone, and in a few cases, it occurs in multiple bones. GSD occurs in a wide range of bones and has been reported to involve the femur, ribs, jaw, humerus, tibia, fibula, and spine (7–9). The clinical symptoms of GSD are diverse, depending on the lesion accumulation and the rate of disease progression. A small number of patients are asymptomatic, and most cases show unspecific symptoms such as pain, swelling, and dysfunction in the affected bones (10–12). The incidence of chylothorax caused by GSD is as high as 17%, and the mortality rate is as high as 34% (13–15). When the cervical spine is involved, it can show spinal cord compression and neurological deficits (5).

Laboratory examinations, radiological examinations and histopathological examinations are crucial for the diagnosis of GSD. Laboratory blood tests can provide evidence to rule out infections, myeloma, hyperparathyroidism, and other diseases, but they are less specific for diagnosis (4, 11). Osteolysis is the most prominent feature of GSD on radiological examinations, which include radiography, computed tomography (CT) scan, magnetic resonance imaging (MRI), emission computed tomography (ECT), and positron emission tomography (PET)/CT (1). Pathological examination is the gold standard for the diagnosis of GSD (4, 9). In 1963, Heffez et al. (16) proposed 8 diagnostic criteria for Gorham-Stout disease, which can provide references for the diagnosis (Table 1). The treatment of GSD includes medical therapy, radiotherapy and surgical intervention or combination therapy (5, 11). In this report, we studied a case of GSD in the right hip joint that was treated with bisphosphonates followed by a total hip arthroplasty.

**Table 1:** Eight criteria proposed by heffez et al. (16) for the diagnosis of Gorham-Stout disease.

	Criteria	Conformity
1	Positive biopsy findings in terms of angiomatous tissue	✓
2	Absence of cellular atypia	✓
3	Minimal or no osteoclastic response and absence of dystrophic calcifications	✓
4	Evidence of local bone progressive resorption	✓
5	Non-expansive lesion	NA
6	Absence of visceral involvement	✓
7	Osteolytic radiographic pattern	✓
8	Negative hereditary, metabolic, neoplastic, immunologic, and infectious etiology	✓

NA, Not available.

## Case report

A 70-year-old man presented with a 10-year history of severe right hip pain and a 2-month history of progressive walking disorder of the lower limbs. The patient was previously treated with nonsteroidal anti-inflammatory drugs elsewhere due to pain, but the pain relief was not significant until the patient was unable to walk and was admitted to the emergency department of our hospital in May 2018. The patient had no history of alcoholism, hormone use, or trauma. Initial physical examination revealed positive tenderness in the right groin, positive patrick's sign, and rotation and abduction significantly less than the left hip joint. No redness or skin temperature increase was observed in the right hip joint.

**Figure 1:**
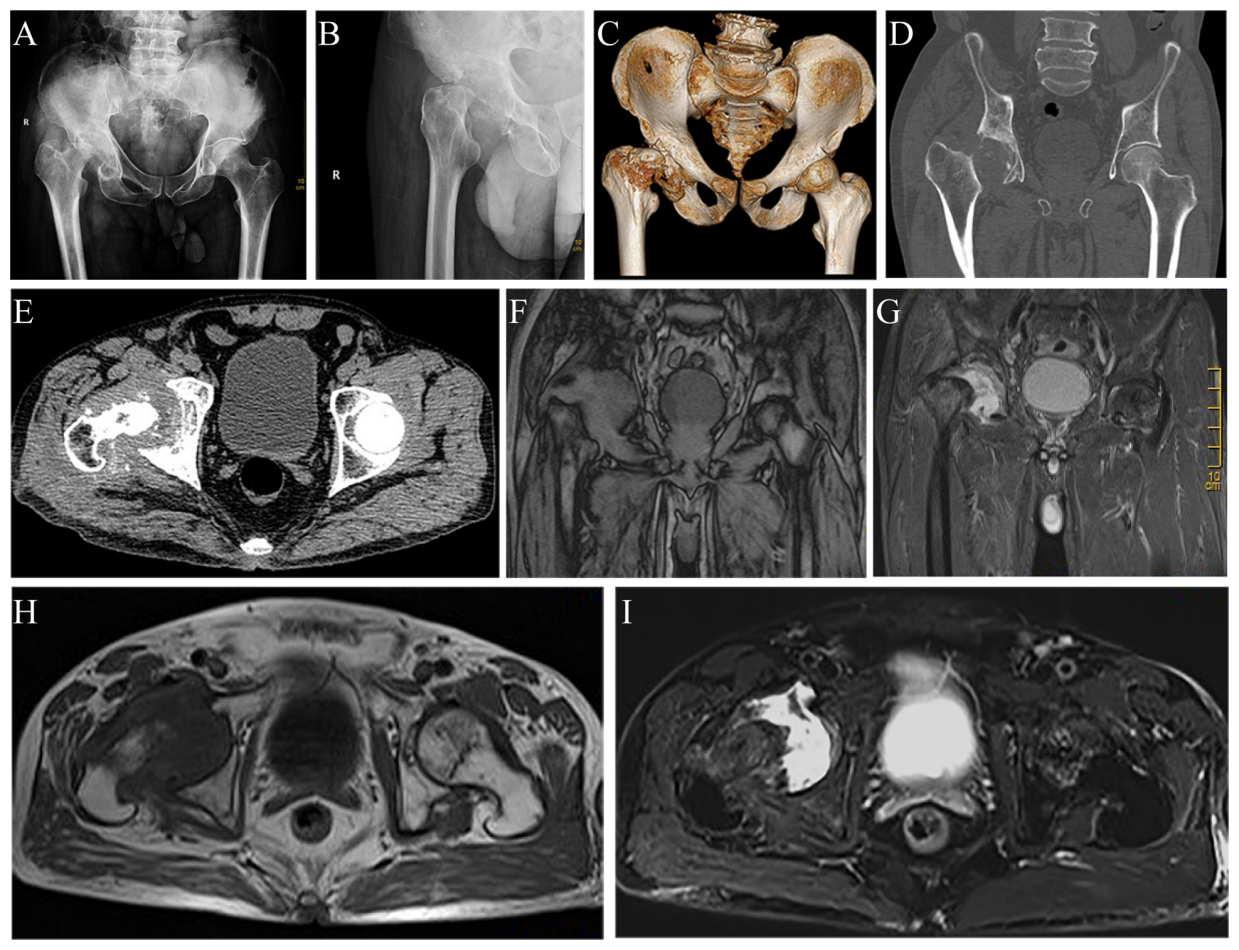
Radiograph images, 3D reconstruction image, CT scan images and MRI images of the hip joint revealing massive osteolysis in the right femoral head, femoral neck, and acetabulum. Anteroposterior (**A**) and lateral (**B**) radiographs of the hip joint show massive osteolysis in the right femoral head, femoral neck, and acetabulum. The 3D reconstruction image (**C**), coronal (**D**), and horizontal (**E**) CT scan images confirm the complete disappearance of the right femoral head and destruction of the femoral neck and acetabulum. Coronal (**F**) and horizontal (**H**) T1-weighted images show extensive hypointense osteolytic lesions involving the right femoral head, femoral neck, and acetabulum. Coronal (**G**) and horizontal (**I**) fat-suppressed T2-weighted images indicate extensive hyperintensity osteolytic lesions.

**Figure 2:**
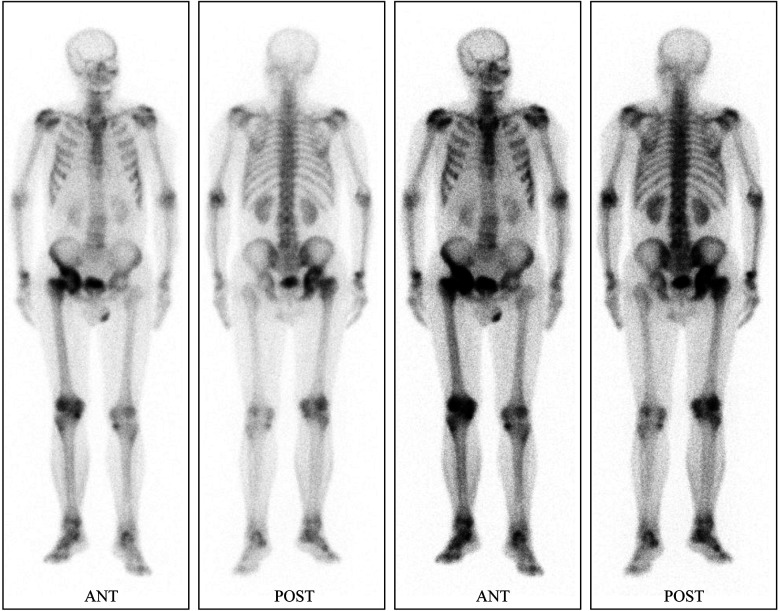
ECT with ^99m^Tc-methylene diphosphate indicates increased focal concentrations of radiotracers in the right femoral head, femoral neck and, acetabulum.

**Figure 3:**
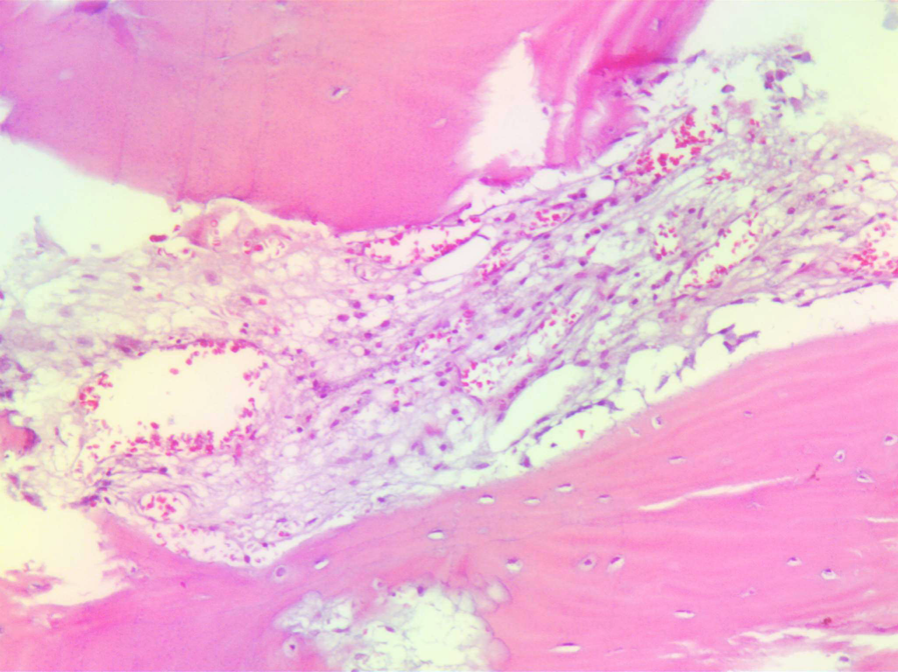
Histopathological examination of a biopsy in the right femur reveals small fragments of destroyed trabeculae with osteoclasts and proliferating thin-walled blood vessels.

**Figure 4:**
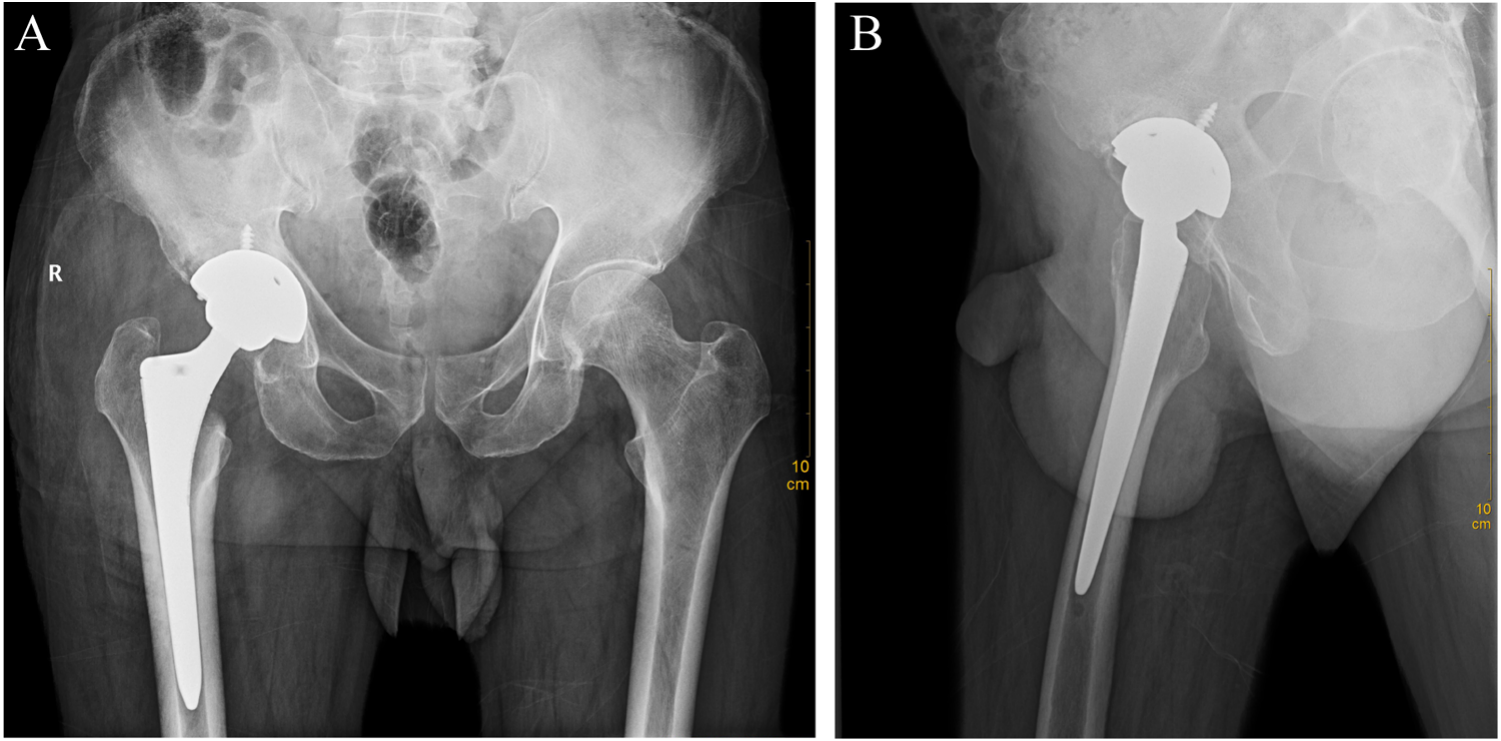
Anteroposterior (**A**) and lateral (**B**) radiographs of the hip joint 3 years after total hip arthroplasty surgery.

Preliminary anteroposterior (Figure 1A) and lateral (Figure 1B) radiograph examinations of the right hip joint indicated that the femoral head had disappeared and that the margins of the femoral neck and acetabulum were incomplete, which were further confirmed by 3D reconstruction images (Figure 1C) and CT scan images (Figures 1D,E). In particular, the 3D reconstruction and CT scan images clearly demonstrated the range of local osteolysis involving the acetabulum and femoral neck. MRI images (Figures 1F–I) showed hypointensity on T1-weighted images, extensive hyperintensity on fat-suppressed T2-weighted images, and massive joint effusion in the right hip joint. To further clarify the progression of the disease, ECT examination (Figure 2) was performed and the results showed increased focal concentrations of radiotracers in the right femoral head, femoral neck, and acetabulum. Therefore, we highly suspected that this patient may be diagnosed with a malignant bone tumor with aggressive osteolytic lesions. In addition, the chest radiograph was normal, and no obvious signs of tuberculosis were found. Laboratory blood tests showed normal levels of leukocytes, neutrophil granulocyte ratio, hypersensitive C-reactive protein, erythrocyte sedimentation rate, serum calcium, phosphorous, alkaline phosphatase, and parathyroid hormone. Oncology indicators such as alpha fetoprotein, carcinoembryonic antigen and PSA were all within the normal range. The T-SPOT test was negative. To further clarify the diagnosis, an open biopsy of the right hip joint was performed under general anesthesia. A total of 6 cm^3^ tissues were obtained from the right femoral neck, synovial membrane, and acetabulum. Subsequent histological examinations showed that the necrotic bone tissue was accompanied by focal aggregation of osteoclasts, giant cells and plasma cells without cellular atypia, some proliferative thin-walled capillaries and lymphatic structures, and replaced by fibrous connective tissue (Figure 3). Combined with clinical manifestations, laboratory blood tests, radiological features and histopathology examinations, the patient met seven out of the eight diagnostic criteria for GSD proposed by Heffez et al. (16) (Table 1). Eventually, the patient was diagnosed with GSD. The patient was then treated with medication, including bisphosphonates (4 mg/week, iv), vitamin D (600 UI/day), and calcium (600 mg/day) for one month. The surgical criteria for total hip replacement in patients with GSD were considered as follows: (i) the patient was 70 years old; (ii) The GSD lesions had involved the acetabulum and the neck of the femur, and in a serious case, the femoral head had completely disappeared. (iii) Contraindications to surgery have been ruled out by relevant examinations. (iii) The patient has a strong need to restore lower limb motor function. We first completely removed the GSD-accumulated bone with the assistance of intraoperative pathologic examination, and then fitted a total hip arthroplasty rather than femoral head arthroplasty to account for the GSD lesions that had accumulated in the acetabulum. To restore the normal walking function of the lower limbs, we performed complete excision of the necrotic tissue followed by total hip arthroplasty. At the 3-year follow-up after surgery, the patient had no pain in the right hip when walking long distances or bearing weight. Radiographs showed that the hip prosthesis was in place and not loose. Moreover, no signs of recurrence were observed in the right hip joint (Figure 4).

## Discussion

GSD is a rare benign osteolytic disease characterized by trabecular destruction and resorption of bone, thin-walled capillaries and lymphatic hyperplasia, and the absence of cellular heterogeneity (1–3). This disease was first reported in 1,838 (17) and later in 1,872 by Jackson (18). Subsequently, Gorham et al. (19) reviewed 24 cases in the literature and named the disease Gorham-Stout disease in 1955. Although this disease was discovered more than 180 years ago, its etiology and pathogenesis are still unknown. Moreover, there is no clear uniform standard for diagnosis and treatment. In our case, GSD involved the right hip joint, leaving the patient unable to walk. At present, there are very few cases of GSD involving the joints. Cases involving the hip joint and leading to complete dissolution and absorption of the femoral head by nontraumatic causes have rarely been reported.

GSD pathology includes osteoclast activation, angiogenesis and lymphatic angiogenesis caused by cytokines such as TGF-*β*, PDGF-BB, and VEGF produced by bone macrophages (4). T cells participate in the activation of osteoclasts through the RANKL/RANK signaling pathway. In addition, the crosstalk between osteoclasts and T cells through the RANKL/RANKR pathway is also involved in the pathological changes of GSD (4). Clinical manifestations, laboratory blood examinations, radiological examinations and histopathological examinations provide strong evidence for the diagnosis of GSD (4, 9, 11). Although some cases of GSD are self-limiting and have no symptoms, the majority of cases progress slowly and the symptoms are nonspecific. Therefore, when the diagnosis of GSD is confirmed, massive osteolysis has formed in the involved bone (9). Therefore, it may not attract the attention of patients and miss the optimal time for treatment of GSD. Insufficient attention or misdiagnosis can lead to missed optimal treatment opportunities for GSD, which can be life-threatening (2). In our case, the patient presented with right hip pain 10 years ago, but the right femoral head had completely disappeared by the time he arrived at our hospital. The results of laboratory blood tests of GSD are usually normal (11, 20). Relevant blood indicators such as serum calcium, phosphorus, alkaline phosphatase, neutrophil ratio, and leukocyte count can help us exclude bacterial and fungal infections, tuberculosis, metabolic diseases (such as renal osteodystrophy), and endocrine diseases (such as hyperparathyroidism) (2, 4, 11). Radiographs are not sensitive enough to identify the early intramedullary or subcortical radiopaque areas in GSD lesion (1). When the course of GSD reaches the second stage and beyond, the radiological examination can reveal the bone atrophy, dissolution, and fragmentation of the affected part until the dissolved bone is completely absorbed and replaced by fibrous connective tissue (21). In contrast, CT scan, 3D reconstruction and MRI imaging can be more sensitive to detect the intramedullary and subcortical lesions of GSD in the first stage (21, 22). CT scan and MRI imaging are usually necessary to determine the range of lesions that require surgical removal. ECT and PET/CT are widely recommended as alternative diagnostic examinations because they can assess bone metabolism to determine GSD progression and the effectiveness of treatment (1, 23). ECT is less costly than PET/CT, but the sensitivity and specificity of ECT in excluding bone tumors and other metabolic diseases are lower than those of PET/CT. Biopsy histopathological examinations are necessary for the diagnosis of GSD in the exclusion of osteosarcoma, metastatic bone tumor, multiple myeloma, and hemangioma.

Medical therapy, radiotherapy, and surgical interventions or combinations are currently available for the treatment of GSD (2, 5, 11). Medical therapy is used to prevent or slow the progression of GSD. The current medical therapy includes bisphosphonates, calcium, vitamin D, steroids, procalcitonin, bevacizumab, and interferon-alpha (5, 24–27). Among them, bisphosphonates are the most widely used to treat GSD. The pharmacological effects of bisphosphonates include inhibition of osteoclast activity, promotion of osteoclast apoptosis, and inhibition of angiogenesis (5, 23, 26, 27). Local administration has fewer side effects, such as gastrointestinal irritation, osteonecrosis of the jaw, and eye inflammation (5, 27). It is recommended to combine calcium and vitamin D as essential basic supplements when using bisphosphonates (11, 23). The course of interferon-alpha treatment for GSD is usually more than 1 year, and it has a wide range of side effects, mainly including blood toxicity, hepatotoxicity and, mental effects (5, 28). It is necessary to closely monitor blood-related indicators during application. The radiotherapy treatment for GSD is controversial. Radiotherapy of 40–45 Gy at 2 Gy per fraction has been reported to effectively inhibit the activity of osteoclasts (29, 30). It may have an inhibitory effect on growth in younger patients. Elderly patients may be unable to tolerate radiotherapy and may even develop radiation-induced tumors. Jose R et al. (31) reported a case of radiotherapy-induced sarcoma in a GSD patient in the left maxilla. Surgical treatment provides the best chance of cure for GSD, especially for patients with massive osteolysis (5, 11, 32). Surgical interventions include resection alone, biological reconstruction using autogenous or allogeneic bone after resection and artificial prosthesis reconstruction after resection (5, 11, 32). Regardless of the surgical approach, complete removal of the osteolytic lesion is a prerequisite to avoid recurrence of GSD. Considering that the GSD lesion had accumulated in the localized the acetabulum and femoral neck and more complete debridement of diseased tissue, we opted for total hip replacement instead of femoral head arthroplasty. Preoperative use of bisphosphonates to prevent disease progression can help control lesion size (2, 11). In the literature, GSD patients with joint involvement treated with artificial prostheses have achieved good results with no recurrence (2, 32). However, there are also risks of loosening, infection, and fractures around the prosthesis. In our case, preoperative use of bisphosphonates to inhibit osteolysis followed by total hip arthroplasty achieved excellent results in restoring hip function.

## Conclusion

GSD is a rare benign osteolytic disease, and its etiology and pathogenesis are still unclear. There are no uniform standards for diagnosis and treatment. The combination of clinical manifestations, laboratory examinations, imaging features, and histopathological characteristics is helpful for diagnosis. Medical therapy, radiotherapy, and surgical interventions or combinations are the options for the treatment of GSD. In our case, the combination of bisphosphonates and artificial prosthesis reconstruction achieved good results in the treatment of GSD involving the hip joint, and there was no recurrence at the 3-year follow-up. Medical therapy combined with artificial prosthesis reconstruction may be an effective treatment approach for GSD.

## Data Availability

The raw data supporting the conclusions of this article will be made available by the authors, without undue reservation.
